# Berberrubine, a Main Metabolite of Berberine, Alleviates Non-Alcoholic Fatty Liver Disease *via* Modulating Glucose and Lipid Metabolism and Restoring Gut Microbiota

**DOI:** 10.3389/fphar.2022.913378

**Published:** 2022-07-08

**Authors:** Sa Yang, Shijie Cao, Congyu Li, Jichao Zhang, Chang Liu, Feng Qiu, Ning Kang

**Affiliations:** ^1^ School of Chinese Materia Medica, Tianjin University of Traditional Chinese Medicine, Tianjin, China; ^2^ State Key Laboratory of Component-based Chinese Medicine, Tianjin University of Traditional Chinese Medicine, Tianjin, China; ^3^ Tianjin State Key Laboratory of Modern Chinese Medicine, Tianjin University of Traditional Chinese Medicine, Tianjin, China; ^4^ State Key Laboratory of Microbial Resources, Institute of Microbiology, Chinese Academy of Sciences, Beijing, China; ^5^ School of Integrative Medicine, Tianjin University of Traditional Chinese Medicine, Tianjin, China

**Keywords:** berberrubine, berberine, NAFLD, glucose and lipid metabolism, gut microbiota

## Abstract

Non-alcoholic fatty liver disease (NAFLD) is a major public health problem in many countries. Berberine (BBR) is an effective therapeutic agent in alleviating NAFLD. Berberrubine (BRB) is one of the main active metabolites of BBR, which shows significant anti-obesity and antihypoglycemic effects. However, whether BRB is responsible for the *in vivo* therapeutic effect and the underlying mechanism of BRB on NAFLD have not been elucidated. In this study, the ability of BRB to ameliorate NAFLD, together with its molecular mechanism, was investigated. The results showed that BRB treatments could significantly improve hepatic steatosis and insulin resistance in high-fat diet (HFD)–fed mice and oleic acid (OA)–treated HepG2 cells. Meanwhile, BBR and BRB treatment similarly prevented lipid accumulation by regulating the protein expression of ATGL, GK, PPARα, CPT-1, ACC1, FAS, and CD36. In addition, compared with BBR, BRB could maintain glucose homeostasis *via* GLUT2, GSK3β, and G6Pase in HFD-fed mice. Furthermore, the components of the gut microbiota in mice were analyzed by 16S rRNA gene sequencing. BBR and BRB treatment could greatly modify the structure and composition of gut microbiota. At the genus level, BBR and BRB treatment decreased *Lactobacillus* and *Romboutsia*, while BBR increased beneficial bacteria, such as *Akkermansia* and *Bacteroides*, and BRB increased beneficial bacteria, such as *Ileibacterium* and *Mucispirillum*. Altogether, both BRB and BBR were active in alleviating NAFLD *in vivo* and BRB might be used as a functional material to treat NAFLD clinically.

## GRAPHICAL ABSTRACT

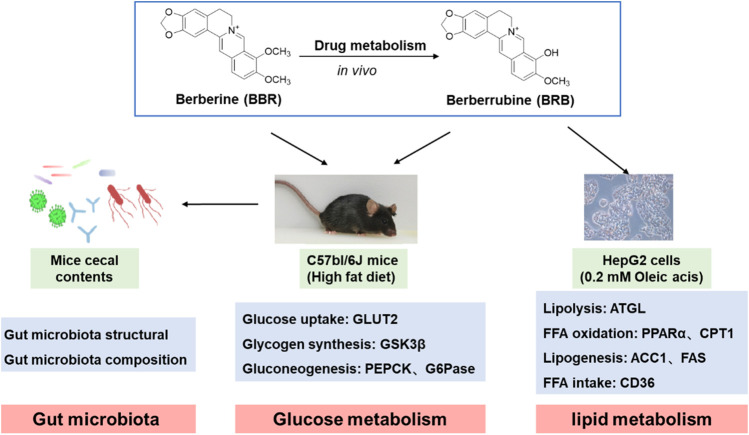

## Introduction

Non-alcoholic fatty liver disease (NAFLD) is characterized by accumulation of fat in the liver, without excessive alcohol consumption and other causes of hepatic steatosis. NAFLD comprises a spectrum of liver diseases from nonalcoholic fatty liver (NAFL) to non-alcoholic steatohepatitis (NASH), and NAFLD could even result in cirrhosis and liver cancer (Watt et al., 2019), which is becoming the leading cause of liver-related morbidity and mortality worldwide (Younossi et al., 2018; Kazankov et al., 2019). NAFLD has become the major cause of chronic liver disease, which is strongly associated with obesity, type 2 diabetes, and other metabolic diseases (Moore et al., 2020).

The pathogenesis of NAFLD is complex and not fully elucidated. Hepatic steatosis is the hallmark feature of NAFLD, which arises from the uptake of fatty acids and *de novo* lipogenesis, surpassing fatty acid oxidation and export (Ipsen et al., 2018). Insulin resistance (IR) is another factor causing the pathogenesis of NAFLD. Numerous studies have demonstrated a strong relationship between lipid metabolism and IR. Improving hepatic IR is a potential therapeutic strategy to treat NAFLD (Zhang et al., 2020). Gut microbiota plays an important role in the pathophysiology of NAFLD through the gut–liver axis (Safari and Gérard, 2019; Judith et al., 2020). It was discovered that gut microbiota contributes not only to the regulation of energy metabolism but also to glucose and lipid homeostasis and is a potential therapeutic target associated with obesity and related metabolic diseases (Cani, 2018). Owing to its complicated pathogenesis, to date, there are few efficient pharmacotherapies available in the market for preventing and treating NAFLD. Botanical natural compounds have become appropriate agents to be explored as ideal treatment options for NAFLD because of the advantages of their wide range of effects and low side effects (Qu et al., 2016).

Berberine (BBR) is an isoquinoline alkaloid isolated from traditional Chinese herbs such as *Coptis Chinensis*. BBR shows anti-obesity and antihypoglycemic effects. However, the bioavailability of BBR is extremely poor (F = 0.36%) (Feng et al., 2019; Feng et al., 2020; Xu X. et al., 2021). In our previous experiments, we found that BBR undergoes extensive metabolism after oral administration, which contributed to the pharmacokinetics–pharmacology disconnection (Zhang et al., 2012; Wang et al., 2017a). Berberrubine (BRB) was one of the main metabolites (65.1%) of BBR in the liver (Tan et al., 2013). BRB showed higher bioavailability (F = 31.6%) than BBR (Wang et al., 2015). Increasing evidence have shown that BRB possesses superior activities such as lipid-lowering (Cao et al., 2013; Zhou et al., 2014), anti-inflammation (Yu et al., 2018), glucose-lowering (Sun et al., 2020), and antioxidation (Pongkittiphan et al., 2015) to BBR. However, whether BRB has beneficial effects on NAFLD and the detailed mechanisms are still not clearly elucidated. In this study, the protective effect of BRB in HFD-fed mice and OA-treated HepG2 cells was evaluated. Also, the underlying mechanism of BRB in regulating NAFLD was elucidated.

## Materials and Methods

### Materials

BBR and BRB (≥98%) were purchased from Shanghai Yuanye Biological Technology Co., Ltd (Shanghai, China). Metformin (MET) (≥98%) and fenofibrate (FEN) (≥98%) were obtained from Shanghai Aladdin Biochemical Technology Co. Ltd and the National Institute for Food and Drug Control, respectively. The mouse insulin ELISA kit was purchased from ZCIBIO Technology Co. Ltd (Shanghai, China). The total cholesterol (TC), triglyceride (TG), high-density lipid-cholesterol (HDL-c), low-density lipid-cholesterol (LDL-c), alanine aminotransferase (ALT), aspartate aminotransferase (AST), urea nitrogen (BUN), and creatinine (CRE) kits were purchased from Nanjing Jiancheng Institute of Biological Engineering (Nanjing, China). Anti–GLUT2 antibody, the primary antibody used for Western blotting, was obtained from Abcam plc (Cambridge, United States). Anti-ACC1, anti-FAS, anti-GSK3β, and anti–p-GSK3β-Ser9 antibodies were purchased from Cell Signaling Technology, Inc. (Beverly, United States). Anti-ATGL was obtained from Santa Cruz Biotechnology Inc. (Santa Cruz, United States). Anti-GK, anti-PPARα, anti-CPT1, anti-CD36, anti-PPARγ, anti-PEPCK, anti-G6Pase, and anti–β-actin were purchased from Proteintech Group, Inc. (Wuhan, China). A secondary antibody was purchased from Beijing Zhongshan Golden Bridge Biotechnology Co., Ltd (Beijing, China).

### Animals and Treatments

**FIGURE 1:**
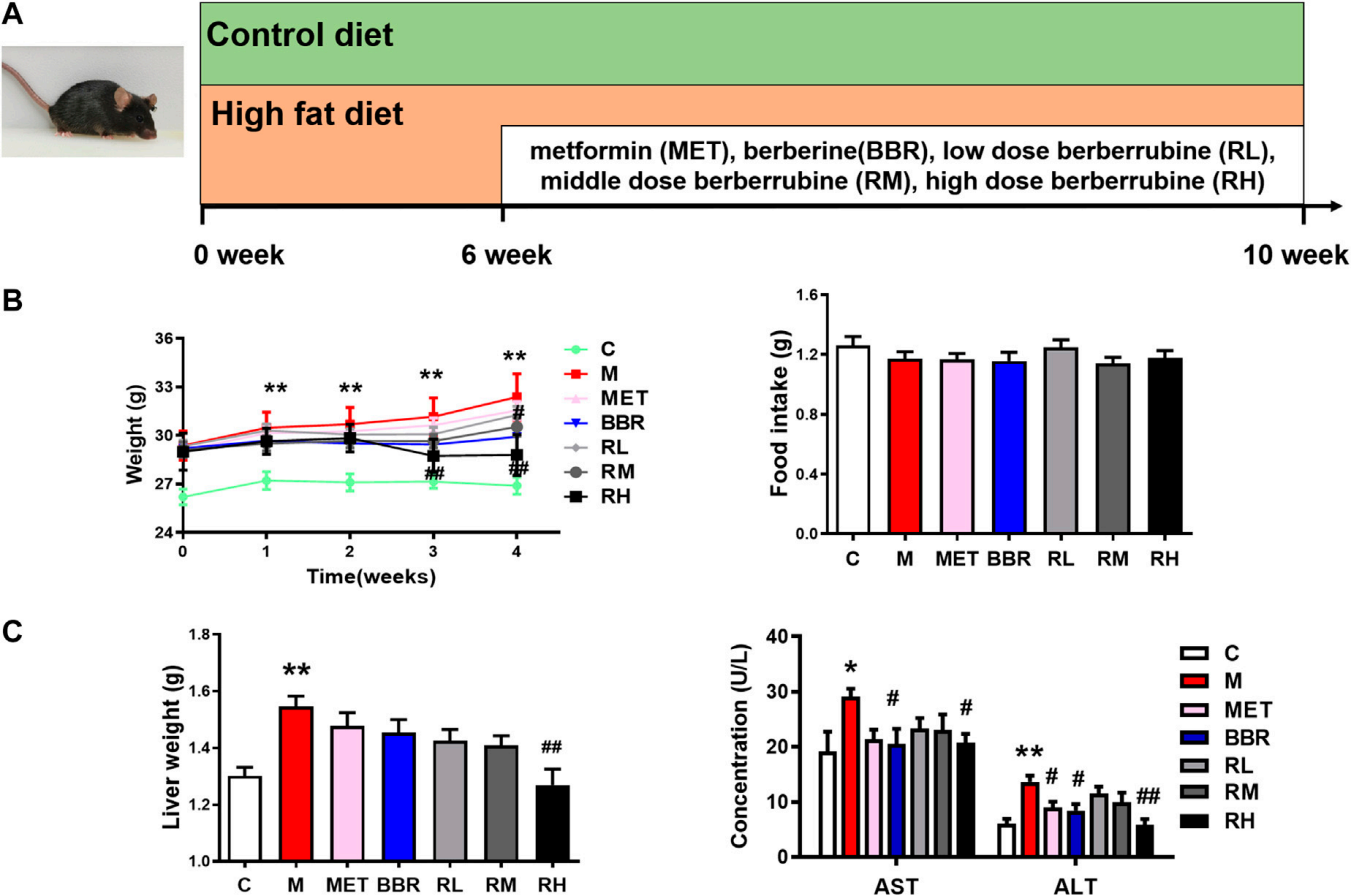
Effect of BRB treatment on body weight, food intake, ALT and AST levels, and liver weight in HFD-fed mice. C57BL/6J mice were fed with HFD chow and treated with MET (40 mg/kg), BBR (40 mg/kg), and BRB (10, 20, and 40 mg/kg) for 4 weeks. MET was the positive control. **(A)** Flow diagram of experimental operation, **(B)** Bodyweights and food intake and **(C)** liver weight and the levels of ALT and AST in the serum of the mice after BBR and BRB treatment. The data were represented as mean ± SEM (n = 8). *p < 0.05 and **p < 0.01, compared with the control group; ^#^ *p* < 0.05 and ^##^ *p* < 0.01, compared with the model group. C, control group; M: model group; MET: metformin group; BBR: berberine group; RL: low-dose berberrubine group; RM: middle-dose berberrubine group; RH: high-dose berberrubine group.

All animal experiments were performed with the approval of the Animal Ethics Committee of Tianjin University of traditional Chinese medicine (approval number: TCM-LAEC2021001). Male C57BL/6J mice weighing 20 ± 2 g (n = 70) were purchased from Vital River Laboratories (Beijing, China). The mice were housed in a temperature- and humidity-controlled facility (temperature 25 ± 2°C, relative humidity 50 ± 5%), with a 12 h light/dark cycle. The mice were given free access to water and diet. After 1 week of acclimation to the environment, the mice were randomly divided into two groups: the control diet group (20% protein, 70% carbohydrate, 10% fat; 3.85 kcal/g; H10010, HFK, China) and the high-fat diet group (20% protein, 20% carbohydrate, 60% fat; 5.24 kcal/g; H10060, China). The NAFLD model was induced in 6-week-old mice fed with HFD for 10 weeks using a protocol from previous studies (Yao et al., 2020; Zamani-Garmsiri et al., 2021). From 6 weeks, 60 mice were randomly divided into six groups: HFD group (M), metformin group (40 mg/kg, MET), BBR group (40 mg/kg, BBR), low-dose BRB (10 mg/kg, RL), middle-dose BRB (20 mg/kg, RM), and high-dose BRB (40 mg/kg, RH). The control group was administered with 0.5% sodium carboxymethyl cellulose (CMC-Na). MET, BBR, and BRB were dissolved in 0.5% CMC-Na solution to prepare a suspension, and the mice were administered once daily with MET, BBR, and BRB by oral gavage for the last 4 weeks (Figure 1A). During the experimental period of 4 weeks, the bodyweight and glucose levels were measured weekly.

Glucose levels were determined with a glucometer (Roche Diagnostics GmbH, Germany) using the samples collected from the tail tip. After the last administration with BBR and BRB, all mice were starved overnight for 12 h with free access to water. Then, the fasting blood samples were collected. Liver, kidney, and cecal content was snap-frozen in liquid nitrogen and then stored at −80°C until analysis.

### Biochemical Parameter Determination and Histological Analysis

The serum was obtained by centrifugation of the blood samples at 3,000 rpm for 15 min at 4°C. The liver tissues were homogenized in absolute ethanol (HDL-c and LDL-c) or PBS (TG and TC) according to a ratio of 1:9. The liver homogenate was centrifuged at 3,000 rpm for 10 min. The levels of serum insulin, ALT, AST, CRE, BUN, TG, TC, LDL-c, and HDL-c were determined using commercial test kits following the manufacturer’s protocol.

The liver was fixed in 4% paraformaldehyde solution, embedded in paraffin wax, cut into 5-μm-thick sections, and stained with hematoxylin–eosin (H&E) reagent according to a standard procedure. Histopathological changes were observed using a fully automated slice scanning system. The NAFLD activity score was evaluated for steatosis, lobular inflammation, and hepatocyte ballooning (Kleiner et al., 2005).

### Intraperitoneal Glucose Tolerance Test (IGTT)

For the glucose tolerance test (GTT), the mice were starved for 12 h and injected with 1 g/kg glucose i.p. The blood samples were obtained from the tail vein and tested at 0, 15, 30, 60, 90, and 120 min after injection. Blood glucose concentration was determined using a glucometer (Roche Diagnostics GmbH, Germany). The curve of glucose concentration over time was plotted, and the area under the curve (AUC) was calculated for each animal.

### Cell Culture and Treatment

Human hepatocellular carcinoma cells (HepG2) were purchased from the Cell Bank of the Chinese Academy of Sciences (Shanghai, China). The HepG2 cells were maintained in a high-glucose DMEM medium containing 10% fetal bovine serum (FBS), 100 U/mL penicillin, and 10 ug/mL streptomycin at 37°C with 5% CO_2_. In all experiments, the cells were cultured to reach 80–90% confluence.

For cell viability assay, the HepG2 cells were plated at a density of 1×10^4^ cells per well in 96-well plates. After 24 h, the HepG2 cells were incubated with different concentrations of BRB (1.25, 2.5, 5, 10, and 20 μM). After 24 h of cell treatment, the media were discarded, and the cells were incubated with MTT solution for 2.5 h. After incubation, the MTT solution in each well was removed, and the formazan product was solubilized in 150 μL DMSO. The absorbance was measured at 490 nm by a microplate reader.

For induction of steatosis, the HepG2 cells were incubated in 0.2 mM oleic acid (OA) medium and treated with different concentrations of BRB (1.25, 2.5, and 5 μM) for 24 h, and 5 μM fenofibrate (FEN) was used as the positive control.

### Oil Red O Staining and Intracellular TG Levels

The HepG2 cells were plated in 6-well plates at 6×10^5^ cells/well. After the cells were fused to 40–60%, the HepG2 cells were treated as described above. After 24 h, the cells were washed three times with PBS and fixed with 4% paraformaldehyde for 30 min at room temperature. Afterward, the cells were washed three times with PBS and stained with 0.5% Oil Red O in isopropanol at room temperature for 10 min. After being washed with PBS three times, the stained lipid droplets were dissolved in isopropanol, and the absorbance was measured at 490 nm to quantify lipid accumulation.

For intracellular TG levels, after treatment with BRB in the presence of OA medium for 24 h, the HepG2 cells were harvested. Intracellular TG levels were measured following the manufacturer’s instructions, and the absorbance was measured at 490 nm. Then, the protein contents in the lysate were determined using the bicinchoninic acid (BCA) kit.

### Glucose Uptake and Glucose Consumption

Glucose uptake assay was performed by using 2-deoxy-2-[(7-nitro-2,1,3-benzoxadiazol-4-yl) amino]- d-glucose (2-NBDG). After BRB treatment, 2-NBDG (50 μM) was added to the cells in serum-free low glucose DMEM medium with 100 nM insulin for 20 min at 37°C and washed twice with PBS. The cells were analyzed and photographed using a high-content system. Glucose consumption in the medium was measured by commercial test kits (Applygen Technologies, Beijing) using the glucose oxidase method by following the manufacturer’s instructions.

### Western Blotting

The tissues or cells were lysed with RIPA lysis buffer (including 1 mM PMSF) for 30 min. The lysates were separated on 8%–12% SDS-PAGE and transferred to PVDF membranes, and then 5% non-fat dry milk was used to block the membranes. The primary antibody was used to incubate the membranes overnight at 4°C. The membranes were washed with TBST (0.1% Tween-20) and incubated with an HRP-conjugated secondary antibody. Protein bands were visualized using the Tanon-5200 system.

### Fecal DNA Extraction and 16S rRNA Sequencing

After 4 weeks of administration of BBR and 40 mg/kg BRB, the cecal contents were collected, snap-frozen, and stored at −80 °C for detection of gut microbiota. Fecal DNA was extracted using the DNA stool Kit. The DNA concentration and molecular weight were evaluated using the NanoDrop one spectrophotometer and agarose gel electrophoresis, respectively. The V3–V4 region of the 16S rRNA gene was amplified by PCR using barcoded primers using TaKaRa Premix Taq^®^ Version 2.0 (TaKaRa Biotechnology Co., Dalian, China). The sequencing libraries were generated in accordance with the standard procedure of the NEBNext^®^ Ultra™ II DNA Library Prep Kit for Illumina^®^ (New England Biolabs, United States) and then sequenced on the NovaSeq6000 platform to generate the paired-end reads. The raw 16S rRNA gene sequence reads were demultiplexed, quality-filtered, merged, and clustered into operational taxonomic units (OTUs) with a 97% similarity cutoff, and the chimeric sequences were identified and removed.

The species complexity within the individual samples and the differences among the samples were indicated by alpha diversity and beta diversity analysis, respectively. Also, the bacterial community difference between the samples was elevated by a principal coordinates analysis (PCoA). Spearman’s correlation analysis was conducted to analyze the relationship between the physiological data and gut microbiota at the genus level in the control, model, BBR, and BRB groups.

### Statistical Analysis

SPSS 19.0 software was used to carry out statistical analysis of the data. The data are expressed as mean ± standard error (MEAN ± SEM). One-way ANOVA followed by the LSD test (homogeneity of variance) or Dunnett’s T3 test (heterogeneity of variance) was performed as appropriate across the data sets. *p* < 0.05 was considered statistically significant.

## Results

### BRB Decreased Hepatic Steatosis in HFD-Fed Mice

The lipid accumulation in the liver is a sign of NAFLD. To assess the effect of BRB on hepatic steatosis, the changes in bodyweight, lipid profiles, and the liver sections in the HFD-fed mice after treatment with BRB were examined. Several studies have found that MET has therapeutic potential to improve NAFLD *via* restoring glucose and lipid metabolic homeostasis (Zhou et al., 2018; Zhang D. et al., 2021). Thus, MET was chosen as a positive control drug in the current work. Compared with the control group, the bodyweight was increased in the HFD group. However, BBR and high-dose BRB blocked the bodyweight gain after 3 and 4 weeks of treatment. Meanwhile, there was no significant difference in food intake, indicating that the decrease in bodyweight was not due to the decrease in food intake (Figure 1B). Compared with the control group, the liver weight, ALT, and AST levels increased significantly in the HFD group. After 4 weeks of administration, BBR decreased the ALT and AST levels and high-dose BRB decreased the body weight, ALT, and AST levels (Figure 1C).

**FIGURE 2:**
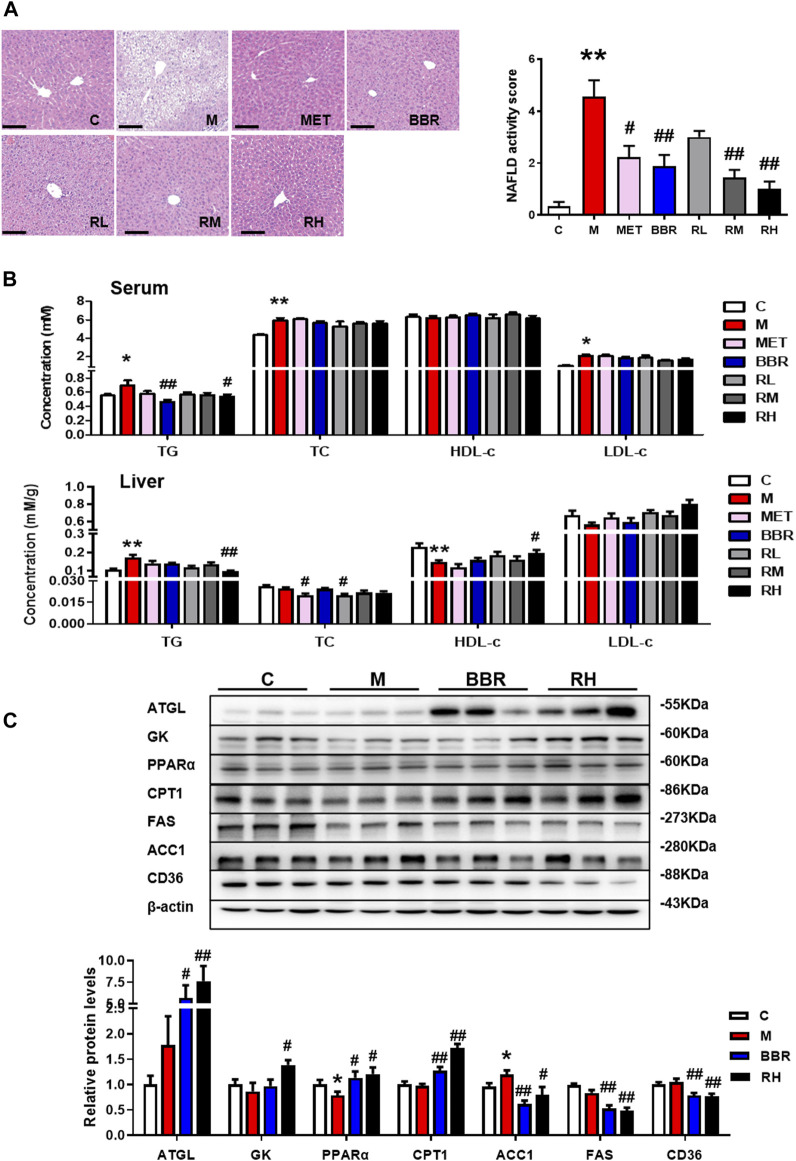
Effect of BRB treatment on hepatic steatosis in HFD-fed mice. C57BL/6J mice were fed with HFD and treated with MET (40 mg/kg), BBR (40 mg/kg), and BRB (10, 20, 40 mg/kg) for 4 weeks. MET was the positive control. **(A)** HE-stained sections of the liver tissues and the NAFLD activity score (Bar = 100 μM). **(B)** Changes in the lipid profile in the serum and liver. **(C)** Expression of proteins associated with lipid metabolism in HFD-fed mice. The data were represented as mean ± SEM (n = 8). *p < 0.05 and **p < 0.01, compared with the control group; ^#^ *p* < 0.05 and ^##^ *p* < 0.01, compared with the model group. C, control group; M: model group; MET: metformin group; BBR: berberine group; RL: low-dose berberrubine group; RM: middle-dose berberrubine group; RH: high-dose berberrubine group.

According to the HE staining, the structure of hepatic lobules was disordered, hepatocytes presented balloon-like swelling, and abnormal lipid droplets were observed in the HFD group, while BBR and BRB treatment restored the structure of liver lobules and reduced balloon-like changes and lipid accumulation. BBR, medium-dose BRB, and high-dose BRB decreased NAFLD activity score (Figure 2A). Furthermore, the levels of lipid profile in the serum and liver tissues were assessed. As shown in Figure 2B, compared with the HFD group, BBR treatment significantly decreased the serum TG levels, while the high-dose BRB treatment significantly reduced the serum TG, hepatic TG, and increased the hepatic HDL-c levels. Altogether, BBR and BRB administration conferred protection against hepatic steatosis in HFD-fed mice, and BRB exerted superior efficacy than BBR in modulating hepatic steatosis in HFD-fed mice.

To investigate the underlying mechanism of BRB against NAFLD, the expression of proteins associated with hepatic lipid metabolism was investigated by using Western blotting. It was revealed that BBR and high-dose BRB treatment enhanced the expression of protein with lipolysis (ATGL), glycerol metabolism (GK), and FFA oxidation (PPAR-α and CPT-1), while suppressing the expression of proteins related to lipogenesis (ACC1 and FAS) and FFA uptake (CD36) in the liver compared with the HFD group (Figure 2C). Consistent with the results of Figure 2B, the suppression of TG acquisition (lipogenesis and FFA uptake) proteins and the promotion of TG removal (lipolysis, glycerol metabolism, and FFA oxidation) proteins were the primary reasons for the reduced TG content with BBR and BRB treatment.

### BRB Ameliorated Glucose Homeostasis in HFD-Fed Mice

**FIGURE 3:**
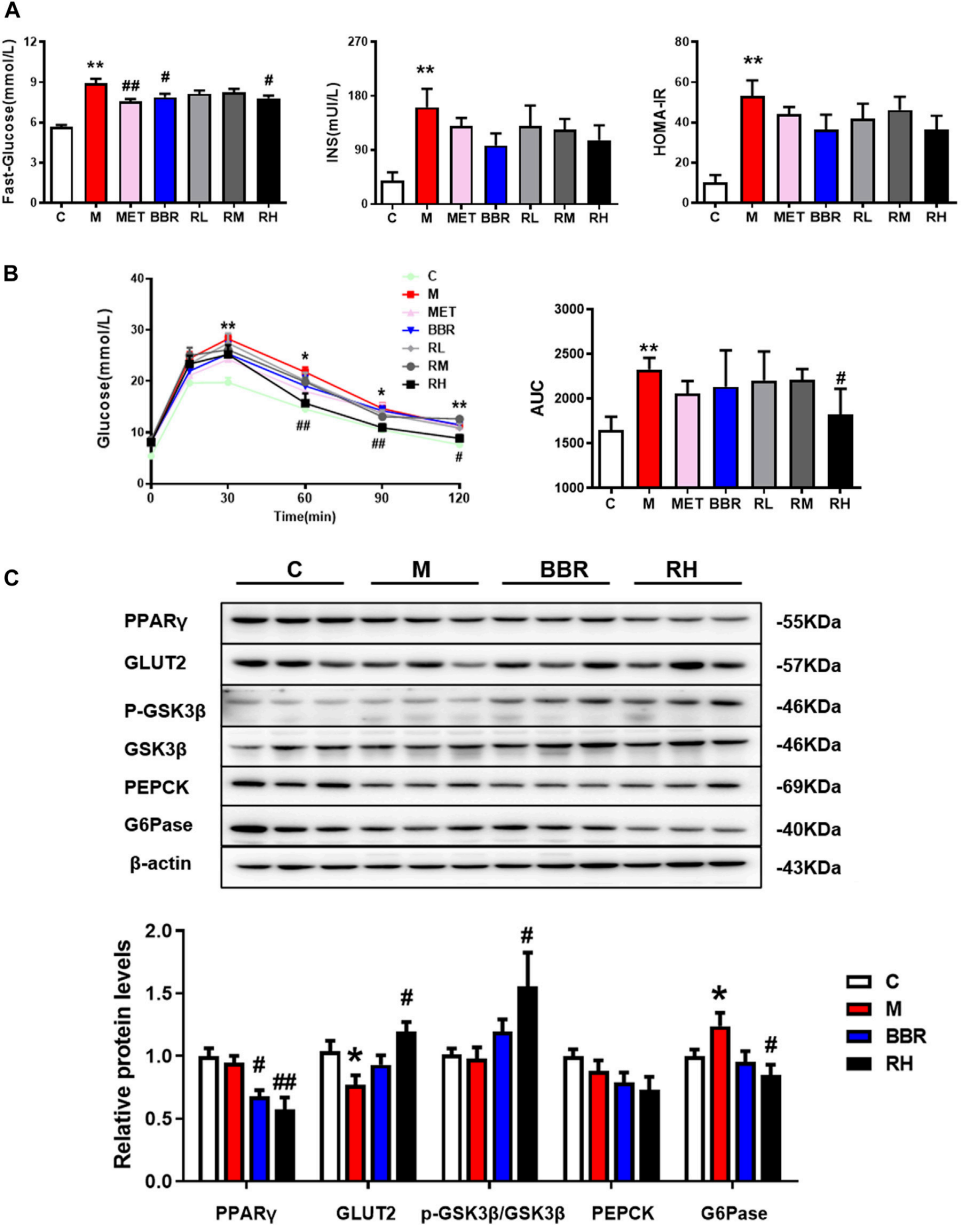
Effect of BRB treatment on glucose homeostasis in HFD-fed mice. C57BL/6J mice were fed with HFD with MET, BBR, and BRB for 4 weeks. MET was the positive control. **(A)** Fasting glucose, insulin levels, and HOMA-IR index. **(B)** Levels of blood glucose and the AUC during IGTT and **(C)** Expression of proteins associated with glucose metabolism in HFD-fed mice. The data were represented as mean ± SEM (n = 8). *p < 0.05 and **p < 0.01, compared with the control group; ^#^ *p* < 0.05 and ^##^ *p* < 0.01, compared with the model group. C, control group; M: model group; MET: metformin group; BBR: berberine group; RL: low-dose berberrubine group; RM: middle-dose berberrubine group; RH: high-dose berberrubine group.

Insulin resistance is regarded as a key factor in the pathogenesis of NAFLD (Birkenfeld and Shulman, 2014). To evaluate the effects of BRB on glucose homeostasis and insulin sensitivity in HFD-fed mice, the levels of glucose and insulin and IGTT were checked after 4 weeks of BRB treatment. As illustrated in Figure 3A, significantly higher glucose and insulin levels were observed in the HFD group than in the control group. Interestingly, the fasting glucose levels were significantly reduced in BBR and high-dose BRB groups. The insulin levels and HOMA-IR index were also reduced in BBR and BRB groups, with no statistical significance. The treatment with high-dose BRB resulted in a significant reduction in blood glucose levels between 0 and 120 min of the IGTT and the AUC value, suggesting that BRB was able to improve glucose tolerance. However, there was no significance in the blood glucose levels and AUC value in the BBR group (Figure 3B). These results suggested that BRB had superior efficacy than BBR in improving glucose homeostasis in HFD-fed mice.

To investigate the underlying mechanism of BRB on glucose lowering effects in HFD-fed mice, the expression of proteins associated with glucose homeostasis was investigated by using Western blotting. It was revealed that BRB treatment suppressed the expression of PPARγ, which was closely related to insulin sensitivity but enhanced the expression of two proteins related to glucose uptake (GLUT2) and glycogen synthesis (p-GSK), while the protein related with gluconeogenesis (G6Pase) in the liver was suppressed compared with the HFD group. Nevertheless, no changes in glucose metabolism–related protein expression were observed in the BBR treatment group (Figure 3C). Thus, these results showed that BRB administration improved glucose homeostasis in HFD mice *via* the promotion of glucose uptake and glycogen synthesis and suppression of gluconeogenesis.

### BRB Decreased Lipid Accumulation in OA-Induced HepG2 Cells

**FIGURE 4:**
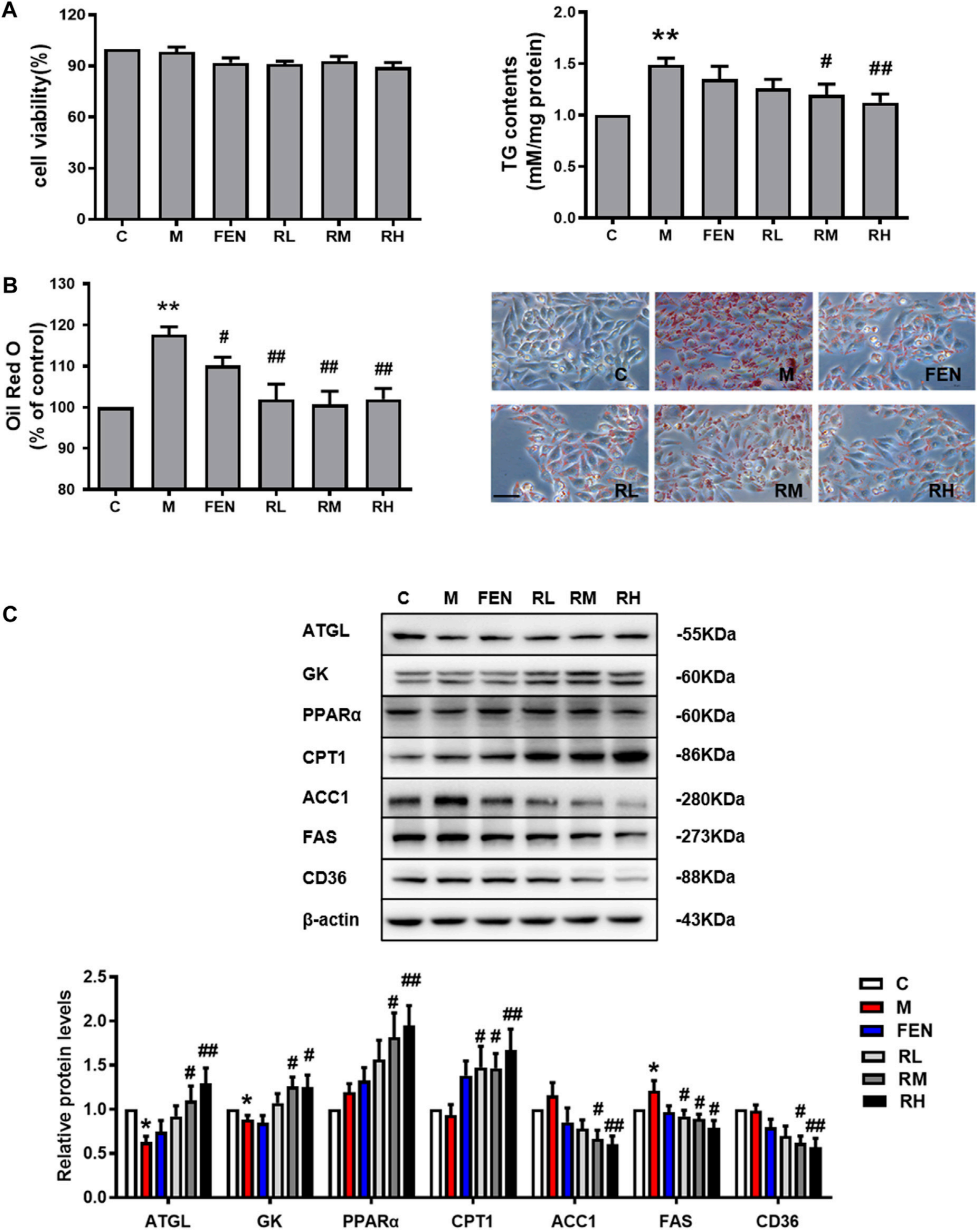
Effect of BRB treatment on hepatic steatosis in OA-HepG2 cells. The HepG2 cells were incubated in 0.2 mM OA and treated with concentrations of BRB (1.25, 2.5, and 5 μM) for 24 h, and FEN (5 μM) was used as the positive control. **(A)** Cell viability and the TG content had different concentrations of BRB and **(B)** the Oil Red O staining (Bar = 50 μM) **(C)** and the expression of proteins are associated with the lipid metabolism in OA-HepG2 cells. The data were represented as mean ± SEM (n = 3). *p < 0.05 and **p < 0.01 compared with the control group; ^#^ *p* < 0.05 and ^##^ *p* < 0.01, compared with the model group. C, control group; M: model group; FEN: fenofibrate group; RL: 1.25 μM berberrubine group; RM: 2.5 μM berberrubine group; RH: 5 μM berberrubine group.

To further investigate the effects of BRB on lipid metabolism, the HepG2 cells were challenged with 0.2 mM OA to induce steatosis. The MTT assay was used to examine the effects of BRB on cell viability. The concentrations of 1.25, 2.5, and 5 μM BRB did not affect cell viability; thus, these concentrations were selected for subsequent studies. Meanwhile, the intracellular TG content was significantly decreased in the 2.5 and 5 μM BRB-treated OA-HepG2 cells (Figure 4A). Subsequently, the lipid accumulation was measured by Oil Red O staining. The results showed that BRB significantly attenuated the OA-induced TG and lipid accumulation in HepG2 cells (Figure 4B). To explore the mechanism of BRB treatment on hepatic steatosis, the expression of proteins associated with lipid metabolism in the HepG2 cells was investigated using Western blotting. The results showed that BRB enhanced protein expression of ATGL, GK, PPAR-α, and CPT-1 in a dose-dependent manner, while suppressing the protein expression of ACC1, FAS, and CD36 in the OA-induced HepG2 cells, compared with the model group (Figure 4C). These results suggested that BRB reduced lipid accumulation in OA-treated HepG2 cells *via* promotion of lipolysis and FFA oxidation and suppression of lipogenesis.

### BRB Ameliorated Glucose Homeostasis in OA-Induced HepG2 Cells

**FIGURE 5:**
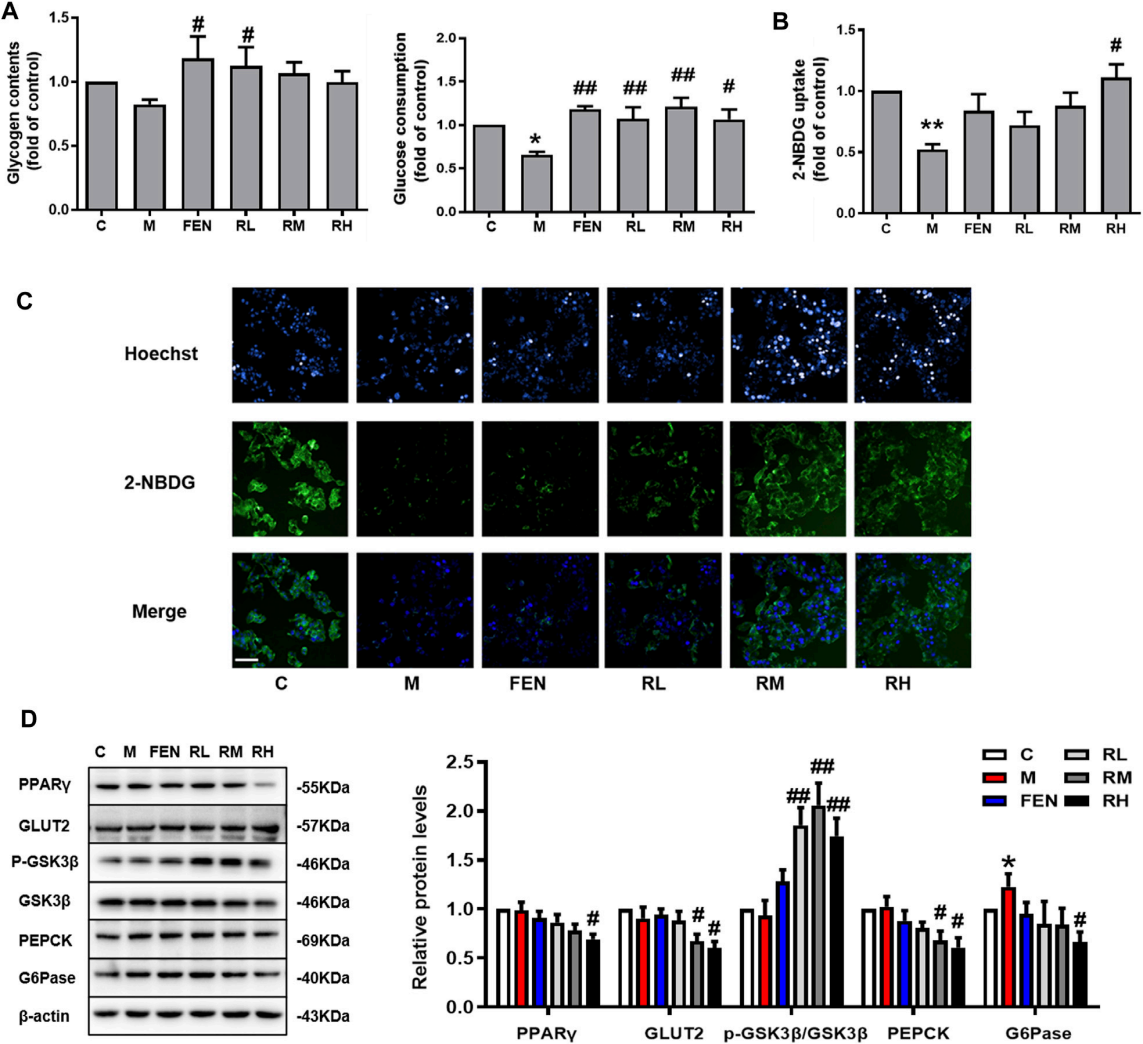
Effect of BRB treatment on glucose homeostasis in OA-treated HepG2 cells. The HepG2 cells were treated with different doses of BRB with 0.2 mM OA for 24 h, and FEN (5 μM) was used as the positive control. **(A)** Glucose consumption and glycogen contents. **(B,C)** 2-NBDG glucose uptake assay (Bar = 50 μM) and **(D)** the expression of proteins associated with glucose homeostasis in OA-HepG2 cells. The data were represented as mean ± SEM (n = 3). *p < 0.05 and **p < 0.01, compared with the control group; ^#^ *p* < 0.05 and ^##^ *p* < 0.01, compared with the model group. C, control group; M: model group; FEN: fenofibrate group; RL: 1.25 μM berberrubine group; RM: 2.5 μM berberrubine group; RH: 5 μM berberrubine group.

To elucidate the effects of BRB on the modulation of glucose metabolism in OA-induced HepG2 cells, the glucose consumption and glycogen contents were measured. A 1.25-μM BRB treatment significantly induced higher glycogen contents and 1.25-, 2.5-, and 5-μM BRB treatment significantly reduced glucose consumption, compared to the model group (Figure 5A). To determine the effect of BRB on glucose uptake, the 2-NBDG glucose uptake assay was performed in OA-treated HepG2 cells. The results showed that the level of fluorescent-labeled glucose significantly decreased in response to OA compared with that of the untreated control cells, and BRB treatment had significantly higher levels of fluorescent-labeled glucose, compared with the model group, indicating that 5 μM BRB significantly improved the glucose uptake (Figure 5B,C).

In addition, the expression of proteins associated with glucose homeostasis in OA-induced HepG2 cells was investigated by using Western blotting. In Figure 5D, BRB treatment enhanced the protein expression of GLUT2 and p-GSK, while suppressing the protein expression of PPARγ, PEPCK, and G6Pase in the liver compared with the model group. Thus, these results suggested that BRB improved glucose homeostasis in OA-stimulated HepG2 cells *via* the promotion of glucose uptake and glycogen synthesis and suppression of gluconeogenesis.

### Impact of BBR and BRB Administration on Gut Microbiota

**FIGURE 6:**
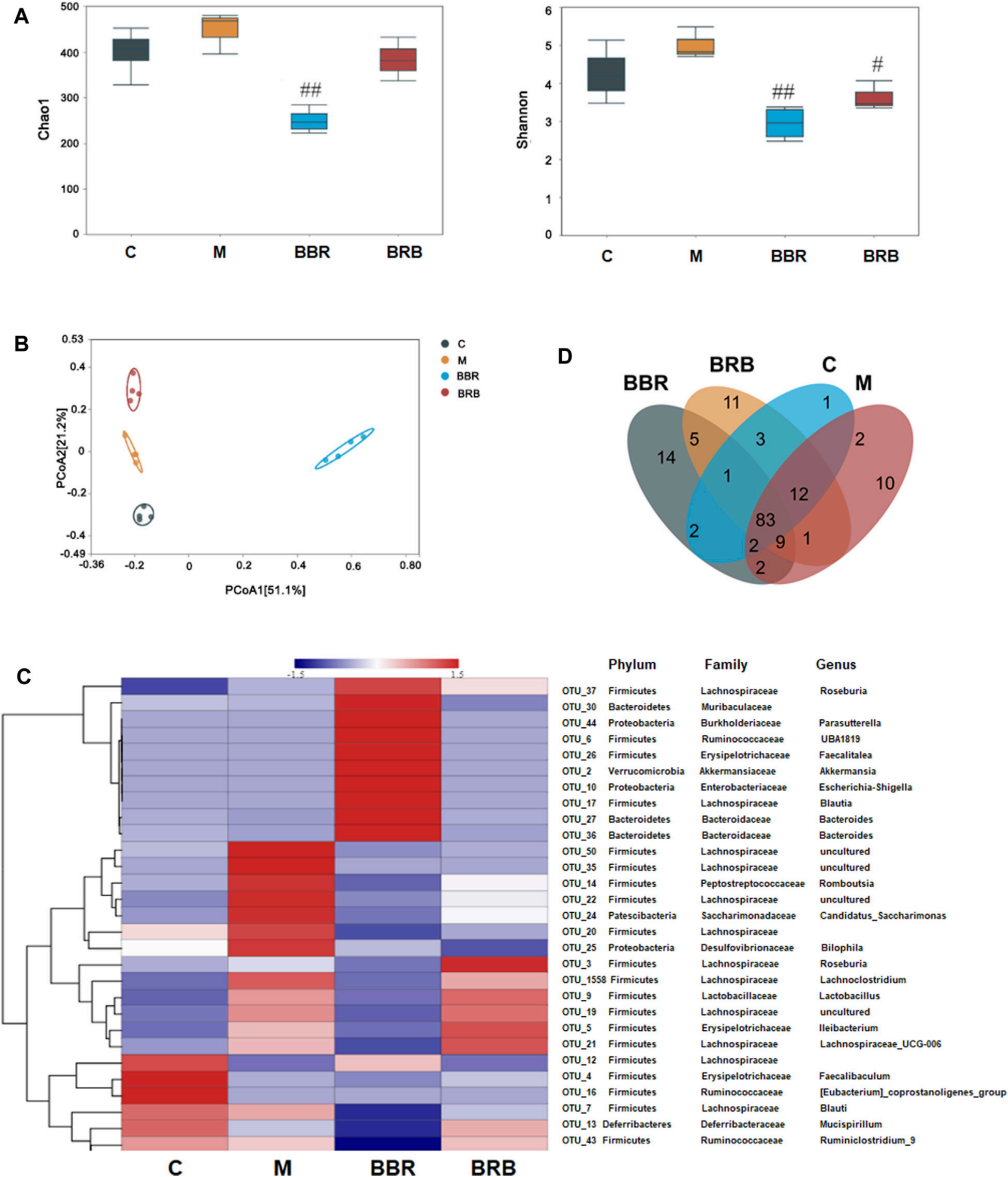
Effect of BBR and BRB treatment on gut microbiota **(A)** Bacteria diversity assay was indicated by Chao index and the Shannon index **(B)** PCoA of the fecal microbial communities in three groups of mice. **(C)** Heatmap showing the relative abundance of classified OTUs. **(D)** Venn diagram. n = 4, C, control group; M: model group; BBR: berberine group; BRB: 40 mg/kg berberrubine group.

The gut microbiota plays a vital role in the development of NAFLD and obesity (Han et al., 2018; Suk and Kim, 2019). The changes in gut microbiota in response to BRB were determined by 16S rRNA sequencing. First, the diversity and richness of gut microbiota, which have been proven to be highly correlated with obesity and metabolic diseases, were analyzed (Chatelier et al., 2013). As shown in Figure 6A based on OTU level, there were no significant differences in Chao1 index and Shannon index between the control and model groups. The Chao1 index and Shannon index were lower in the BBR group than in model groups. The Chao1 index showed no significant difference between 40 mg/kg BRB and model groups; whereas the Shannon index was lower in the 40 mg/kg BRB group than in the model group. These results indicated that BBR and BRB treatment changed the richness and diversity of gut microbiota community. PCoA showed an entire separation between the four communities, indicating that there was a significant difference in gut microbial composition between the treatment and model groups (Figure 6B).

To better understand the effect of BRB treatment on gut microbiota composition, the bacterial communities in the four groups were analyzed. A heatmap with a hierarchical cluster was produced, and the relative abundance of intestinal bacteria was analyzed at phylum, family, and genus levels. The hierarchical cluster indicated that BBR and BRB treatment could significantly change the composition of gut microbiota (Figure 6C). A Venn diagram was used to show the shared and unique genus (Figure 6D). BBR and BRB shared 98 genera. There were 18 and 27 unique genera in the BBR and BRB group, respectively, which suggested that there was similarity and difference in the composition of the gut microbiota between the BBR and BRB group.

### Effects of BBR and BRB Treatment on the Gut Microbial Community Structure

**FIGURE 7:**
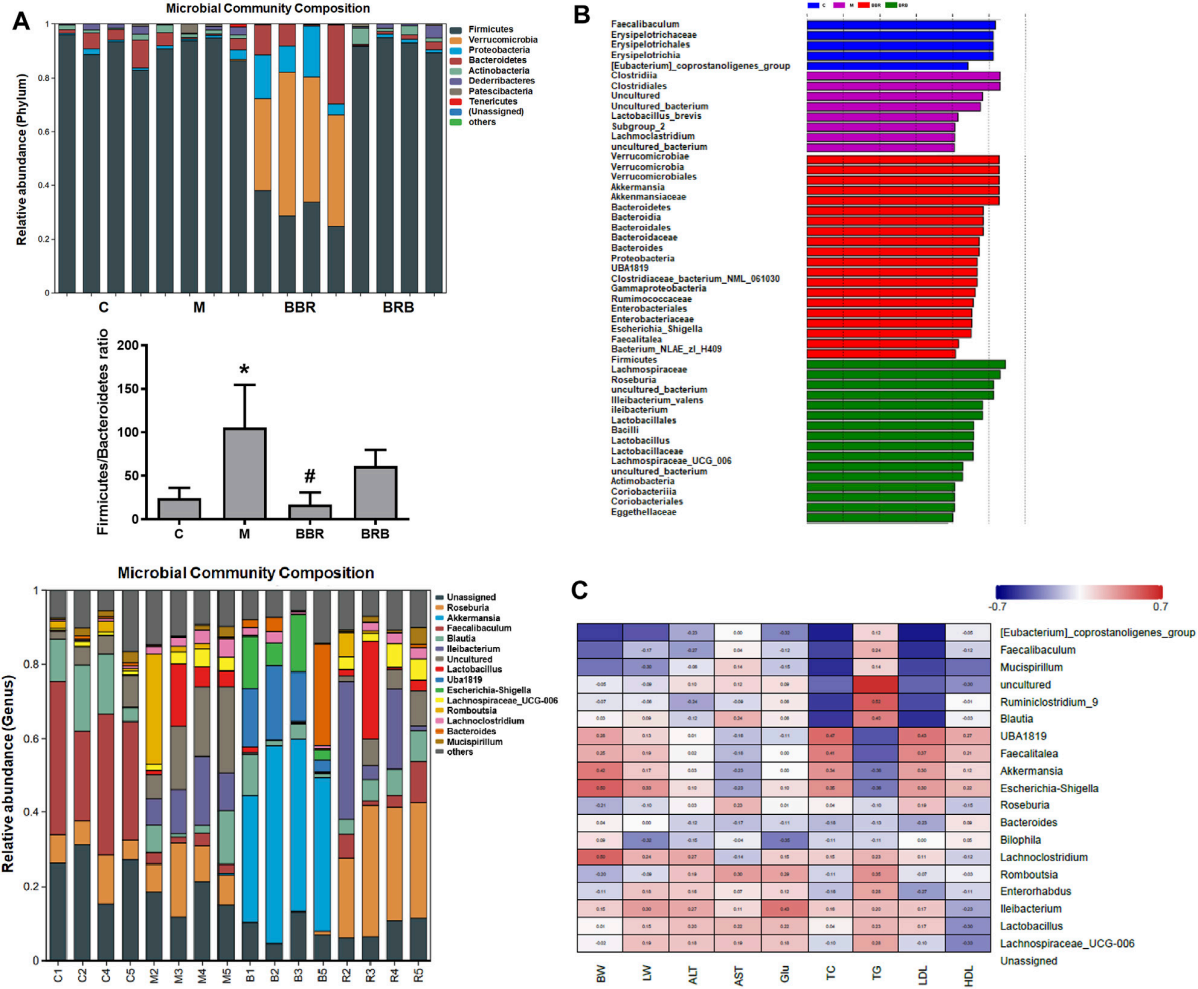
Effects of BBR and BRB treatment on the gut microbial community structure at the genus level in HFD-fed mice. **(A)** Microbial community bar plot at phylum and genus levels. **(B)** Lefse analysis and **(C)** heatmap of the Spearman correlation between the alterations in gut microbial population and the changes in host parameters related to bodyweight (BW), liver weight (LW), AST, ALT, Glu, TC, TG, LDL-c, and HDL-c levels. Negative (blue) or positive (red) Spearman’s correlation was observed. The data were represented as mean ± SEM (n = 4). *p < 0.05, compared with the control group; ^#^ *p* < 0.05, compared with the model group. C, control group; M: model group; BBR: berberine group; BRB: 40 mg/kg berberrubine group.

At the phylum levels, the ratio of *Firmicutes* and *Bacteroidetes* has been an important marker reflecting microbiota changes, which is positively correlated with NAFLD (Nakano H et al., 2020). In this study, the ratio of Firmicutes/Bacteroidetes markedly increased in the model group, whereas it decreased in both BBR and BRB groups (Figure 7A). At the genus levels, the results showed that the relative abundances of *Faecalibaculum*, *Blautia*, and *Mucispirillum* decreased, but those of *Ileibacterium*, Lachnospiraceae*_UCG-006*, *Lactobacillus*, and *Lachnoclostridium* increased in the model group compared with the control group. BBR and 40 mg/kg BRB similarly decreased the abundance of *Romboutsia* and *Lactobacillus*. In addition, BBR increased beneficial bacteria, such as *Akkermansia* and *Bacteroides*, and decreased harmful bacteria, such as Lachnospiraceae*_UCG-006* and *Ileibacterium*. Meanwhile, 40 mg/kg BRB increased the abundance of beneficial bacteria, such as *Roseburia* and *Mucispirillum* (Figure 7A). Furthermore, linear discriminant analysis (LDA) (Figure 7B) confirmed the discriminative features of the abovementioned results. Overall, these results indicated that BBR and BRB treatment reversed HFD-induced gut microbiota dysbiosis.

Finally, a heatmap of Spearman correlations to further explore the relationships between the gut microbiome and NAFLD biomarkers was generated (Figure 7C). The results suggested that *Mucispirillum*, *Akkermansia*, *Escherichia-Shigella*, *Lachnoclostridium*, *Romboutsia*, *Ileibacterium*, and *Lactobacillus* correlated with ALT, AST, glucose, TC, TG, LDL-c, and HDL-c, respectively, indicating that these gut microbiotas might play a central role in the amelioration of liver lipid and glucose dysfunction. BBR may affect the levels of biomarkers *via* modulating *Lactobacillus*, *Romboutsia*, and *Akkermansia* and 40 mg/kg BRB may affect the levels of biomarkers by modulating *Lactobacillus*, *Romboutsia*, *Roseburia*, and *Mucispirillum*, respectively.

## Discussion

BBR, a principal bioactive component of *Coptis chinensis*, commonly used for treating bacterial diarrhea, is an effective therapeutic agent for alleviating NAFLD as proven by many researchers in various animal models and patients (Chang et al., 2016; Luo et al., 2019; Zhu et al., 2019). There is an ongoing phase 4 clinical trial (NCT0398572) of BBR to evaluate its curative effect on NAFLD (Li et al., 2021). Previous studies suggested that BBR might alleviate NAFLD by modulating the glucose and lipid metabolism and restoring gut microbiota (Cao et al., 2016; Zhao et al., 2017). However, our previous studies demonstrated that the absolute bioavailability of BBR was very low due to insufficient absorption and extensive metabolism. In human and animal models, the main primary metabolites of BBR are BRB, thalifendine, demethyleneberberine, and jatrorrhizine, which have multiple pharmacological activities (Qiang et al., 2016; Wang et al., 2017b; Wang C. et al., 2021), suggesting that the metabolites of BBR may contribute to its bioactivities. BRB is predominantly lipophilic and could be absorbed more efficiently than BBR (Spinozzi et al., 2014) and has been proven to possess lipid-lowering, glucose-lowering, and anti-inflammatory effects (Cao et al., 2013; Zhou et al., 2014; Yu et al., 2018; Sun et al., 2021). The effects of BBR on NAFLD have been well-described; however, little is known about the effects of BRB on NAFLD.

Our data showed that BRB significantly reduced bodyweight gain, liver, and serum TG content, ATL, AST, and fasting glucose levels and improved glucose tolerance in HFD-fed mice. In addition, BRB also attenuated TG content and lipid droplet and increased glucose uptake and glucose computation in OA-induced HepG2 cells. Notably, compared with BBR, BRB significantly decreased blood glucose levels and the AUC value in the IGTT. In addition, BRB showed a stronger inhibitory effect on the hepatic TG contents than BBR. These results demonstrated that BRB, the main metabolite of BBR, was a potential therapeutic agent in the treatment of NAFLD and exerted superior efficacy to BBR in attenuating NAFLD.

Hepatic steatosis is the major pathogenic factor in NAFLD, which results from excessive lipolysis, *de novo* lipogenesis, FFA uptake, and decreased FFA oxidation. There are numerous evidence demonstrating that BBR can decrease hepatic steatosis in HepG2 cells (Ren et al., 2020), HFD-fed mice (Sun et al., 2018), and patients with NAFLD (Chang et al., 2016). The beneficial effects of BBR appear to be partially mediated by activating AMPK or upregulating LDLR expression (Kong et al., 2004). In our previous study, we found that BRB also could exert TG-lowering effects in HepG2 cells *via* AMPK and LDLR (Cao et al., 2013b). However, the beneficial effects of BRB *in vivo* were unclear, and the underlying mechanism remains incompletely understood. The current results showed that BBR and BRB treatment significantly upregulated the expression of the protein related to lipolysis (ALGL) and fatty acid β-oxidation (CPT-1 and PPAR-α), while markedly reducing the expression of protein related with *de novo* lipogenesis (ACC1 and FAS) and fatty acid translation (CD36). These results showed that BBR and BRB have a similar mechanism for lipid metabolism, indicating that BRB is the active metabolite of BBR, and both play a role in improving lipid metabolism.

Insulin resistance is a key factor in the pathophysiology of NAFLD. Glucose and insulin stimulate hepatic *de novo* lipogenesis in individuals with NAFLD (Smith et al., 2020). Enhanced gluconeogenesis and glucose uptake and decreased glycogen synthesis are the hallmarks of hepatic insulin resistance (Saltiel and Kahn, 2001). Increasing evidence suggests that BBR can improve insulin resistance, and several potential mechanisms have been discovered. PPARγ agonists are insulin-sensitizing agents; however, the overactivation of PPARγ may induce weight gain and steatosis in patients and animals. It has been reported that inhibition of PPARγ activity can also improve insulin sensitivity (Ge et al., 2015). Some studies showed that BBR increased PPARγ expression in the liver (Rafiei et al., 2019) but decreased PPARγ expression in the liver in other studies (Zhou et al., 2008; Wang L. et al., 2021). In our study, BBR decreased PPARγ expression in the HFD-fed mice liver. In addition, BRB could also inhibit the PPARγ expression in HFD-fed mice liver and OA-induced HepG2 cells. On the other hand, BRB significantly reduced the protein level of gluconeogenesis (PEPCK and G6Pase) and enhanced the protein expression of glucose uptake (GLUT2) and glycogen synthesis (GSK3β) both in HFD-fed mice and in OA-induced HepG2 cells, but BBR did not affect the expression of GLUT2, PEPCK, G6Pase, and GSK3β *in vivo*. These data demonstrated that aside from BBR, BRB could improve glucose homeostasis by modulating the expression of the proteins related to glucose metabolism *in vivo*, indicating that BRB contributed to the glucose-lowering property of BBR.

Multiple studies have shown that the pathogenesis of NAFLD is closely associated with gut microbiota (Hu et al., 2020). Gut microbiota composition modulates glucose homeostasis and hepatic lipid metabolism, contributing to NAFLD development (Schoeler and Caesar, 2019). BBR was transformed into an absorbed metabolite BRB by gut microflora (Wang et al., 2017b; Zhang ZW. et al., 2021), which was the important material basis for the bioactivities of BBR. Herein, BBR and BRB treatment changed the diversity and structure of the imbalanced gut microbiota community, which were consistent with other results (Zhang et al., 2015; Li et al., 2019). The specific taxonomical identification suggested that BBR and BRB may have directly modulated the abundance of some intestinal bacteria. BBR and BRB shared 98 genera, but they have 18 and 27 unique genera, respectively, which suggested that there were both some similarities and differences in the composition of the gut microbiota between the BBR and BRB group. BBR and BRB similarly decreased *Romboutsia*, which was obesity-related bacteria, and *Lactobacillus*, which may decrease insulin sensitivity and increase inflammation. In addition, BBR increased beneficial bacteria, such as *Akkermansia* and *Bacteroides*, which were reported to improve insulin resistance and reduce inflammation, and decreased harmful bacteria, such as Lachnospiraceae*_UCG-006* and *Ileibacterium*, which were potentially related to obesity and inflammation and BRB increased beneficial bacteria, such as *Roseburia* and *Mucispirillum*, which produce short-chain fatty acids. Altogether, the benefits of BBR and its metabolite, BRB, on hypoglycemic and hypolipidemic activities may be partly attributed to gut bacteria. Meanwhile, there were different changes in gut microbiota after treatment with BBR and BRB, which might induce the different effects of BBR and BRB against NAFLD.

High-dose BRB but not BBR reduces the hepatic TG and increases the hepatic HDL-c levels; these results may be due to different effects of BBR and BRB on gut microbiota. BRB particularly increased *Roseburia* and *Mucispirillum*, which was closely related to TG and HDL-c (Li et al., 2020; Xu D. et al., 2021). Gut microbiota can regulate NAFLD through several mechanisms, including changing the permeability of the intestine, producing harmful metabolites, and altering the expression of genes (Ma et al., 2017). Whether these mechanisms were associated with the therapeutic effect of BBR and its metabolite BRB on NAFLD remains unclear, which deserves further studies.

## Conclusion

BRB, the major metabolites of BBR, showed a stronger effect on improving hepatic steatosis and insulin resistance in NAFLD models. Mechanically, BRB could modulate protein expression related to glucose and lipid metabolism. Furthermore, the changes in gut microbiota composition were associated with the therapeutic effects of BRB on NAFLD. These findings suggested that BRB was the active form of BBR to alleviate NAFLD *in vivo*, indicating that BRB, the metabolite of BBR, contributed to the therapeutic effects of BBR on NAFLD. BRB has the potential to be further developed into a promising candidate for the treatment of NAFLD.

## Data Availability

The datasets presented in this study can be found in online repositories. The names of the repository/repositories and accession number(s) can be found below: https://www.ncbi.nlm.nih.gov/, PRJNA831773.

## Glossary

2-NBDG: 2-deoxy-2-[(7-nitro-2,1,3-benzoxadiazol-4-yl)amino]-d-glucose
ACC1: acetyl-CoA carboxylase 1
ALT: alanine aminotransferase
AST: aspartate aminotransferase
ATGL: adipose triglyceride lipase
BBR: berberine
BRB: berberrubine
BUN: urea nitrogen
CD36: fatty acid translocase
CMC-Na: sodium carboxymethyl cellulose
BCA: bicinchoninic acid
CPT-1: carnitine acyl transferase 1
CRE: creatinine
FAS: fatty acid synthase
FBS: fetal bovine serum
FEN: fenofibrate
G6Pase: glucose-6-phosphatase
GK: glycerokinase
GLUT2: glucose transporter 2
GSK3β: glycogen synthase kinase-3β
HDL-c: high-density lipid-cholesterol
HepG2: human hepatocellular carcinoma cells
HFD: high-fat diet
IGTT: intraperitoneal glucose tolerance test
LDL-c: low-density lipid-cholesterol
MET: metformin
NAFLD: non-alcoholic fatty liver disease
OA: oleic acid
PEPCK: phosphoenolpyruvate carboxykinase
PPARα: peroxisome proliferator–activated receptor α
PPARγ: peroxisome proliferator–activated receptor γ
TC: total cholesterol
TG: triglyceride

## References

[B1] Aron-Wisnewsky J. Warmbrunn M. V. Nieuwdorp M. Clément K. (2020). Nonalcoholic Fatty Liver Disease: Modulating Gut Microbiota to Improve Severity? Gastroenterology 158 (7), 1881–1898. 10.1053/j.gastro.2020.01.049 32044317

[B2] Birkenfeld A. L. Shulman G. I. (2014). Nonalcoholic Fatty Liver Disease, Hepatic Insulin Resistance, and Type 2 Diabetes. Hepatology 59 (2), 713–723. 10.1002/hep.26672 23929732 PMC3946772

[B3] Cani P. D. (2018). Human Gut Microbiome: Hopes, Threats and Promises. Gut 67 (9), 1716–1725. 10.1136/gutjnl-2018-316723 29934437 PMC6109275

[B4] Cao S. Zhou Y. Xu P. Wang Y. Yan J. Bin W. (2013). Berberine Metabolites Exhibit Triglyceride-Lowering Effects via Activation of AMP-Activated Protein Kinase in Hep G2 Cells. J. Ethnopharmacol. 149 (2), 576–582. 10.1016/j.jep.2013.07.025 23899453

[B5] Cao S. Xu P. Yan J. Liu H. Liu L. Cheng L. (2018). Berberrubine and its Analog, Hydroxypropyl‐berberrubine, Regulate LDLR and PCSK9 Expression via the ERK Signal Pathway to Exert Cholesterol‐lowering Effects in Human Hepatoma HepG2 Cells. J. Cell Biochem. 120, 1340–1349. 10.1002/jcb.27102 30335889

[B6] Cao Y. Pan Q. Cai W. Shen F. Chen G. Y. Xu L. M. (2016). Modulation of Gut Microbiota by Berberine Improves Steatohepatitis in High-Fat Diet-Fed BALB/C Mice. Arch. Iran. Med. 19 (3), 197–203. 10.161903/AIM.008 26923892

[B7] Chang X. Wang Z. Zhang J. Yan H. Bian H. Xia M. (2016). Lipid Profiling of the Therapeutic Effects of Berberine in Patients with Nonalcoholic Fatty Liver Disease. J. Transl. Med. 14, 266. 10.1186/s12967-016-0982-x 27629750 PMC5024486

[B17] Chatelier E. L. Nielsen T. Qin J. Prifti E. Hildebrand F. Falony G. (2013). Richness of Human Gut Microbiome Correlates with Metabolic Markers. Nature 500 (7464), 541–546. 10.1038/nature12506 23985870

[B8] Feng X. Sureda A. Jafari S. Memariani Z. Tewari D. Annunziata G. (2019). Berberine in Cardiovascular and Metabolic Diseases: From Mechanisms to Therapeutics. Theranostics 9 (7), 1923–1951. 10.7150/thno.30787 31037148 PMC6485276

[B9] Feng X. Wang K. Cao S. Ding L. Qiu F. (2020). Pharmacokinetics and Excretion of Berberine and its Nine Metabolites in Rats. Front. Pharmacol. 11, 594852. 10.3389/fphar.2020.594852 33584274 PMC7874128

[B10] Ge Z. Zhang P. Hong T. Tang S. Meng R. Bi Y. (2015). Erythropoietin Alleviates Hepatic Insulin Resistance via PPARγ-dependent AKT Activation. Sci. Rep. 5, 17878. 10.1038/srep17878 26643367 PMC4672330

[B11] Han R. Ma J. Li H. (2018). Mechanistic and Therapeutic Advances in Non-Alcoholic Fatty Liver Disease by Targeting the Gut Microbiota. Front. Med. 12 (6), 645–657. 10.1007/s11684-018-0645-9 30178233

[B12] Hu H. Lin A. Kong M. Yao X. Yin M. Xia H. (2020). Intestinal Microbiome and NAFLD: Molecular Insights and Therapeutic Perspectives. J. Gastroenterol. 55 (2), 142–158. 10.1007/s00535-019-01649-8 31845054 PMC6981320

[B13] Ipsen D. H. Lykkesfeldt J. Tveden-Nyborg P. (2018). Molecular Mechanisms of Hepatic Lipid Accumulation in Non-Alcoholic Fatty Liver Disease. Cell Mol. Life Sci. 75 (18), 3313–3327. 10.1007/s00018-018-2860-6 29936596 PMC6105174

[B14] Kazankov K. Jørgensen S. M. D. Thomsen K. L. Møller H. J. Vilstrup H. George J. (2019). The Role of Macrophages in Nonalcoholic Fatty Liver Disease and Nonalcoholic Steatohepatitis. Nat. Rev. Gastroenterol. Hepatol. 16 (3), 145–159. 10.1038/s41575-018-0082-x 30482910

[B15] Kleiner D. E. Brunt E. M. Van Natta M. Behling C. Contos M. J. Cummings O. W. (2005). Design and Validation of a Histological Scoring System for Nonalcoholic Fatty Liver Disease. Hepatology 41 (6), 1313–1321. 10.1002/hep.20701 15915461

[B16] Kong W. Wei J. Abidi P. Lin M. Inaba S. Li C. (2004). Berberine Is a Novel Cholesterol-Lowering Drug Working through a Unique Mechanism Distinct from Statins. Nat. Med. 10 (12), 1344–1351. 10.1038/nm1135 15531889

[B18] Li Q. P. Dou Y. X. Huang Z. W. Chen H. B. Li Y. C. Chen J. N. (2021). Therapeutic Effect of Oxyberberine on Obese Non-Alcoholic Fatty Liver Disease Rats. Phytomedicine 85, 153550. 10.1016/j.phymed.2021.153550 33831691

[B19] Li X. Xiao Y. Song L. Huang Y. Chu Q. Zhu S. (2020). Effect of Lactobacillus Plantarum HT121 on Serum Lipid Profile, Gut Microbiota, and Liver Transcriptome and Metabolomics in a High-Cholesterol Diet-Induced Hypercholesterolemia Rat Model. Nutrition 79-80, 110966. 10.1016/j.nut.2020.110966 32942130

[B20] Li Y. Li J. Su Q. Liu Y. (2019). Sinapine Reduces Non-Alcoholic Fatty Liver Disease in Mice by Modulating the Composition of the Gut Microbiota. Food Funct. 10 (6), 3637–3649. 10.1039/c9fo00195f.PMID.31165837 31165837

[B21] Luo Y. Tian G. Zhuang Z. Chen J. You N. Zhuo L. (2019). Berberine Prevents Non-Alcoholic Steatohepatitis-Derived Hepatocellular Carcinoma by Inhibiting Inflammation and Angiogenesis in Mice. Am. J. Transl. Res. 11 (5), 2668–2682. 31217846 PMC6556646

[B22] Ma J. Zhou Q. Li H. (2017). Gut Microbiota and Nonalcoholic Fatty Liver Disease: Insights on Mechanisms and Therapy. Nutrients 9 (10), 1124. 10.3390/nu9101124 PMC5691740 29035308

[B23] Moore M. P. Cunningham R. P. Dashek R. J. Mucinski J. M. Rector R. S. (2020). A Fad Too Far? Dietary Strategies for the Prevention and Treatment of NAFLD. Obes. (Silver Spring) 28 (10), 1843–1852. 10.1002/oby.22964 PMC7511422 32893456

[B24] Nakano H. Wu S. Sakao K. Hara T. He J. Garcia S. (2020). Bilberry Anthocyanins Ameliorate NAFLD by Improving Dyslipidemia and Gut Microbiome Dysbiosis. Nutrients 12 (11), 3252. 10.3390/nu12113252 PMC7690841 33114130

[B25] Pongkittiphan V. Chavasiri W. Supabphol R. (2015). Antioxidant Effect of Berberine and its Phenolic Derivatives against Human Fibrosarcoma Cells. Asian Pac J. Cancer Prev. 16 (13), 5371–5376. 10.7314/apjcp.2015.16.13.5371 26225680

[B26] Qiang X. Xu L. Zhang M. Zhang P. Wang Y. Wang Y. (2016). Demethyleneberberine Attenuates Non-Alcoholic Fatty Liver Disease with Activation of AMPK and Inhibition of Oxidative Stress. Biochem. Biophys. Res. Commun. 472 (4), 603–609. 10.1016/j.bbrc.2016.03.019 26970305

[B27] Qu L. L. Yu B. Li Z. Jiang W. X. Jiang J. D. Kong W. J. (2016). Gastrodin Ameliorates Oxidative Stress and Proinflammatory Response in Nonalcoholic Fatty Liver Disease through the AMPK/Nrf2 Pathway. Phytother. Res. 30 (3), 402–411. 10.1002/ptr.5541 26634892

[B28] Rafiei H. Omidian K. Bandy B. (2019). Dietary Polyphenols Protect against Oleic Acid-Induced Steatosis in an *In Vitro* Model of NAFLD by Modulating Lipid Metabolism and Improving Mitochondrial Function. Nutrients 11 (3), 541. 10.3390/nu11030541 PMC6471211 30832407

[B29] Ren G. Guo J. H. Qian Y. Z. Kong W. J. Jiang J. D. (2020). Berberine Improves Glucose and Lipid Metabolism in HepG2 Cells through AMPKα1 Activation. Front. Pharmacol. 11, 647. 10.3389/fphar.2020.00647 32457629 PMC7225256

[B30] Safari Z. Gérard P. (2019). The Links between the Gut Microbiome and Non-alcoholic Fatty Liver Disease (NAFLD). Cell Mol. Life Sci. 76 (8), 1541–1558. 10.1007/s00018-019-03011-w 30683985 PMC11105223

[B31] Saltiel A. R. Kahn C. R. (2001). Insulin Signalling and the Regulation of Glucose and Lipid Metabolism. Nature 414 (6865), 799–806. 10.1038/414799a 11742412

[B32] Schoeler M. Caesar R. (2019). Dietary Lipids, Gut Microbiota and Lipid Metabolism. Rev. Endocr. Metab. Disord. 20 (4), 461–472. 10.1007/s11154-019-09512-0 31707624 PMC6938793

[B33] Smith G. I. Shankaran M. Yoshino M. Schweitzer G. G. Chondronikola M. Beals J. W. (2020). Insulin Resistance Drives Hepatic De Novo Lipogenesis in Nonalcoholic Fatty Liver Disease. J. Clin. Invest. 130 (3), 1453–1460. 10.1172/JCI134165 31805015 PMC7269561

[B34] Spinozzi S. Colliva C. Camborata C. Roberti M. Ianni C. Neri F. (2014). Berberine and its Metabolites: Relationship between Physicochemical Properties and Plasma Levels after Administration to Human Subjects. J. Nat. Prod. 77 (4), 766–772. 10.1021/np400607k 24593257

[B35] Suk K. T. Kim D. J. (2019). Gut Microbiota: Novel Therapeutic Target for Nonalcoholic Fatty Liver Disease. Expert Rev. Gastroenterol. Hepatol. 13 (3), 193–204. 10.1080/17474124.2019.1569513 30791767

[B36] Sun R. Kong B. Yang N. Cao B. Feng D. Yu X. (2021). The Hypoglycemic Effect of Berberine and Berberrubine Involves Modulation of Intestinal Farnesoid X Receptor Signaling Pathway and Inhibition of Hepatic Gluconeogenesis. Drug Metab. Dispos. 49 (3), 276–286. 10.1124/dmd.120.000215 33376148

[B37] Sun R. Kong B. Yang N. Cao B. Feng D. Yu X. (2021). The Hypoglycemic Effect of Berberine and Berberrubine Involves Modulation of Intestinal Farnesoid X Receptor Signaling Pathway and Inhibition of Hepatic Gluconeogenesis. Drug Metab. Dispos. 49 (3), 276–286. 10.1124/dmd.120.000215 33376148

[B38] Sun Y. Xia M. Yan H. Han Y. Zhang F. Hu Z. (2018). Berberine Attenuates Hepatic Steatosis and Enhances Energy Expenditure in Mice by Inducing Autophagy and Fibroblast Growth Factor 21. Br. J. Pharmacol. 175 (2), 374–387. 10.1111/bph.14079 29065221 PMC5758394

[B39] Tan X. S. Ma J. Y. Feng R. Ma C. Chen W. J. Sun Y. P. (2013). Tissue Distribution of Berberine and its Metabolites after Oral Administration in Rats. PLoS One 8 (10), e77969. 10.1371/journal.pone.0077969 24205048 PMC3815028

[B40] Wang C. Cheng Y. Zhang Y. Jin H. Zuo Z. Wang A. (2021a). Berberine and its Main Metabolite Berberrubine Inhibit Platelet Activation through Suppressing the Class I PI3Kβ/Rasa3/Rap1 Pathway. Front. Pharmacol. 12, 734603. 10.3389/fphar.2021.734603 34690771 PMC8531212

[B41] Wang K. Chai L. Feng X. Liu Z. Liu H. Ding L. (2017a). Metabolites Identification of Berberine in Rats Using Ultra-high Performance Liquid Chromatography/quadrupole Time-Of-Flight Mass Spectrometry. J. Pharm. Biomed. Anal. 139, 73–86. 10.1016/j.jpba.2017.02.038 28279930

[B42] Wang K. Feng X. Chai L. Cao S. Qiu F. (2017b). The Metabolism of Berberine and its Contribution to the Pharmacological Effects. Drug Metab. Rev. 49 (2), 139–157. 10.1080/03602532.2017.1306544 28290706

[B43] Wang L. Xu B. Sagada G. Ng W. K. Chen K. Zhang J. (2021b). Dietary Berberine Regulates Lipid Metabolism in Muscle and Liver of Black Sea Bream (Acanthopagrus Schlegelii) Fed Normal or High-Lipid Diets. Br. J. Nutr. 125 (5), 481–493. 10.1017/S0007114520003025 32718379

[B44] Wang X. Wang S. Ma J. Ye T. Lu M. Fan M. (2015). Pharmacokinetics in Rats and Tissue Distribution in Mouse of Berberrubine by UPLC-MS/MS. J. Pharm. Biomed. Anal. 115 (115), 368–374. 10.1016/j.jpba.2015.07.031 26279368

[B45] Watt M. J. Miotto P. M. De Nardo W. Montgomery M. K. (2019). The Liver as an Endocrine Organ-Linking NAFLD and Insulin Resistance. Endocr. Rev. 40 (5), 1367–1393. 10.1210/er.2019-00034 31098621

[B46] Xu D. Feng M. Chu Y. Wang S. Shete V. Tuohy K. M. (2021a). The Prebiotic Effects of Oats on Blood Lipids, Gut Microbiota, and Short-Chain Fatty Acids in Mildly Hypercholesterolemic Subjects Compared with Rice: A Randomized, Controlled Trial. Front. Immunol. 12, 787797. 10.3389/fimmu.2021.787797 34956218 PMC8697019

[B47] Xu X. Yi H. Wu J. Kuang T. Zhang J. Li Q. (2021b). Therapeutic Effect of Berberine on Metabolic Diseases: Both Pharmacological Data and Clinical Evidence. Biomed. Pharmacother. 133 (4), 110984. 10.1016/j.biopha.2020.110984 33186794

[B48] Yao C.-C. Tong Y.-X. Jiang H. Yang D.-R. Zhang X.-J. Zhang P. (2020). Native Polypeptide Vglycin Prevents Nonalcoholic Fatty Liver Disease in Mice by Activating the AMPK Pathway. J. Funct. Foods 73, 104110. 10.1016/j.jff.2020.104110

[B49] Younossi Z. Anstee Q. M. Marietti M. Hardy T. Henry L. Eslam M. (2018). Global Burden of NAFLD and NASH: Trends, Predictions, Risk Factors and Prevention. Nat. Rev. Gastroenterol. Hepatol. 15 (1), 11–20. 10.1038/nrgastro.2017.109 28930295

[B50] Yu X. T. Xu Y. F. Huang Y. F. Qu C. Xu L. Q. Su Z. R. (2018). Berberrubine Attenuates Mucosal Lesions and Inflammation in Dextran Sodium Sulfate-Induced Colitis in Mice. Plos One 13 (3), e0194069. 10.1371/journal.pone.0194069 29538417 PMC5851626

[B51] Zamani-Garmsiri F. Hashemnia S. M. R. Shabani M. Bagherieh M. Emamgholipour S. Meshkani R. (2021). Combination of Metformin and Genistein Alleviates Non-Alcoholic Fatty Liver Disease in High-Fat Diet-Fed Mice. J. Nutr. Biochem. 87, 108505. 10.1016/j.jnutbio.2020.108505 32956824

[B52] Zhang C. H. Zhou B. G. Sheng J. Q. Chen Y. Cao Y. Q. Chen C. (2020). Molecular Mechanisms of Hepatic Insulin Resistance in Nonalcoholic Fatty Liver Disease and Potential Treatment Strategies. Pharmacol. Res. 159, 104984. 10.1016/j.phrs.2020.104984 32502637

[B53] Zhang D. Ma Y. Liu J. Deng Y. Zhou B. Wen Y. (2021a). Metformin Alleviates Hepatic Steatosis and Insulin Resistance in a Mouse Model of High-Fat Diet-Induced Nonalcoholic Fatty Liver Disease by Promoting Transcription Factor EB-dependent Autophagy. Front. Pharmacol. 12, 689111. 10.3389/fphar.2021.689111 34366846 PMC8346235

[B54] Zhang J. Zhou F. Lu M. Ji W. Niu F. Zha W. (2012). Pharmacokinetics-pharmacology Disconnection of Herbal Medicines and its Potential Solutions with Cellular Pharmacokinetic-Pharmacodynamic Strategy. Curr. Drug Metab. 13, 558–576. 10.2174/1389200211209050558 22475335

[B55] Zhang X. Zhao Y. Xu J. Xue Z. Zhang M. Pang X. (2015). Modulation of Gut Microbiota by Berberine and Metformin during the Treatment of High-Fat Diet-Induced Obesity in Rats. Sci. Rep. 5 (5), 14405. 10.1038/srep14405 26396057 PMC4585776

[B56] Zhang Z. W. Cong L. Peng R. Han P. Ma S. R. Pan L. B. (2021b). Transformation of Berberine to its Demethylated Metabolites by the CYP51 Enzyme in the Gut Microbiota. J. Pharm. Anal. 11 (5), 628–637. 10.1016/j.jpha.2020.10.001 34765276 PMC8572679

[B57] Zhao L. Cang Z. Sun H. Nie X. Wang N. Lu Y. (2017). Berberine Improves Glucogenesis and Lipid Metabolism in Nonalcoholic Fatty Liver Disease. BMC Endocr. Disord. 17 (1), 13. 10.1186/s12902-017-0165-7 28241817 PMC5329945

[B58] Zhou J. Massey S. Story D. Li L. (2018). Metformin: An Old Drug with New Applications. Int. J. Mol. Sci. 19 (10). 10.3390/ijms19102863 PMC6213209 30241400

[B59] Zhou J. Y. Zhou S. W. Zhang K. B. Tang J. L. Guang L. X. Ying Y. (2008). Chronic Effects of Berberine on Blood, Liver Glucolipid Metabolism and Liver PPARs Expression in Diabetic Hyperlipidemic Rats. Biol. Pharm. Bull. 31 (6), 1169–1176. 10.1248/bpb.31.1169 18520050

[B60] Zhou Y. Cao S. Wang Y. Xu P. Yan J. Bin W. (2014). Berberine Metabolites Could Induce Low Density Lipoprotein Receptor Up-Regulation to Exert Lipid-Lowering Effects in Human Hepatoma Cells. Fitoterapia 92, 230–237. 10.1016/j.fitote.2013.11.010 24321576

[B61] Zhu X. Bian H. Wang L. Sun X. Xu X. Yan H. (2019). Berberine Attenuates Nonalcoholic Hepatic Steatosis through the AMPK-SREBP-1c-SCD1 Pathway. Free Radic. Biol. Med. 141, 192–204. 10.1016/j.freeradbiomed.2019.06.019 31226399

